# Mouse Pulmonary Adenoma Susceptibility 1 Locus Is an Expression QTL Modulating *Kras*-4A

**DOI:** 10.1371/journal.pgen.1004307

**Published:** 2014-04-17

**Authors:** Alice Dassano, Francesca Colombo, Gaia Trincucci, Elisa Frullanti, Antonella Galvan, Angela Pettinicchio, Loris De Cecco, Andrea Borrego, Olga Célia Martinez Ibañez, Tommaso A. Dragani, Giacomo Manenti

**Affiliations:** 1 Department of Predictive and Preventive Medicine, Fondazione IRCCS, Istituto Nazionale dei Tumori, Milan, Italy; 2 Laboratory of Immunogenetics, Instituto Butantan, Saõ Paulo, Brazil; National Cancer Institute, United States of America

## Abstract

Pulmonary adenoma susceptibility 1 (*Pas1*) is the major locus responsible for lung tumor susceptibility in mice; among the six genes mapping in this locus, *Kras* is considered the best candidate for *Pas1* function although how it determines tumor susceptibility remains unknown. In an (A/J×C57BL/6)F4 intercross population treated with urethane to induce lung tumors, *Pas1* not only modulated tumor susceptibility (LOD score = 48, 69% of phenotypic variance explained) but also acted, in lung tumor tissue, as an expression quantitative trait locus (QTL) for *Kras*-4A, one of two alternatively spliced *Kras* transcripts, but not *Kras*-4B. Additionally, *Kras*-4A showed differential allelic expression in lung tumor tissue of (A/J×C57BL/6)F4 heterozygous mice, with significantly higher expression from the A/J-derived allele; these results suggest that *cis*-acting elements control *Kras*-4A expression. In normal lung tissue from untreated mice of the same cross, *Kras*-4A levels were also highly linked to the *Pas1* locus (LOD score = 23.2, 62% of phenotypic variance explained) and preferentially generated from the A/J-derived allele, indicating that *Pas1* is an expression QTL in normal lung tissue as well. Overall, the present findings shed new light on the genetic mechanism by which *Pas1* modulates the susceptibility to lung tumorigenesis, through the fine control of *Kras* isoform levels.

## Author Summary

A person's risk of developing cancer depends on both genetic and environmental factors. To study the genetic predisposition to cancer without the influence of environmental variables, scientists study mice treated with urethane, a chemical carcinogen that induces lung tumors. By crossing inbred (genetically identical) strains of mice that are either resistant or susceptible to urethane-induced cancer, researchers can search for genes associated with tumor formation in the offspring. From previous work of this type using second-generation mice, it was already known that a region on chromosome 6 was associated with tumor formation. Now, a new study, carried out in a fourth-generation mouse population, focused to a single gene of chromosome 6 called *Kras*. This gene forms two different messenger RNA transcripts, called *Kras*-4A and *Kras*-4B, that produce two proteins with slightly different structure and, perhaps, function. The study found that mice susceptible to lung tumors have relatively more *Kras*-4A messenger RNA than resistant mice and that this difference may be due to small variations in the DNA near or within this gene.

## Introduction

The Pulmonary adenoma susceptibility 1 (*Pas1*) locus, mapping in the distal region of chromosome 6, is the major modulator of lung tumor susceptibility in mice [1], [2]. In inbred mouse strains, the *Pas1* locus has a conserved haplotype consisting of six genes clustered in a ∼450-kb region [3]. From proximal to distal, these genes are branched chain aminotransferase 1 (*Bcat1*), lymphoid-restricted membrane protein *(Lrmp)*, cancer susceptibility candidate 1 *(Casc1)*, LYR motif containing 5 *(Lyrm5)*, *v*-Ki-ras2 Kirsten rat sarcoma viral oncogene homolog *(Kras)* and intermediate filament tail domain containing 1 (*Ifltd1*). Four of these genes, namely *Lrmp*, *Casc1*, *Kras* and *Ifltd1*, have been singled out as candidate genes for *Pas1* locus functions in fine-mapping studies [4]–[6]. Although for none of these genes is there a clear demonstration of a role in lung tumor susceptibility, there is substantial evidence supporting the involvement of *Kras* as the key effector of the *Pas1* locus.

*Kras* encodes a small GTPase that functions as a molecular switch in signal transduction, influencing cell proliferation [7]. Permanently activating mutations, at codons 12, 13 and 61, are frequently found in both spontaneous and chemically induced lung tumors in mice [8], [9]. Moreover, in mouse models in which mutant *Kras* can be activated by somatic recombination in the lung, animals are highly susceptible to lung tumorigenesis and develop multiple lung tumors at 100% incidence with a short latency [10], [11]. Nonetheless, as heterozygous *Kras* knockout mice have higher susceptibility to chemically induced lung tumorigenesis than wild-type mice, the wild-type *Kras* allele may have a tumor suppression function [12]. The double role of *Kras* in lung carcinogenesis—tumor suppressor when wild-type and oncogene when mutated—led to the hypothesis that lung cancer susceptibility could result from the subtle balance between expression levels of wild-type and mutated *Kras* [13]. Given the frequent occurrence of activating *Kras* mutations in mouse lung tumors, an increase in *Kras* gene expression in lung tumor tissue could be expected to raise the level of active *Kras* protein, thereby providing the growth advantage characteristic of neoplasms. Evidence supporting this mechanism was provided by the observation that, in mice, *Kras* mRNA levels in normal lung tissue were ∼2-fold higher in strains susceptible to lung tumorigenesis (both highly susceptible A/J mice and intermediate-susceptible FVB/N and 129/Sv mice) than in a resistant strain (C57BL/6) [13].

Our understanding of the mode of action of *Kras* in lung tumorigenesis is further complicated by the existence of two main transcripts, namely *Kras*-4A and -4B, generated by alternative splicing of its fourth coding exon. The two transcripts differ in their 3′-termini and give rise to two proteins (of 189 and 188 residues, respectively) with different C-terminal sequences. Because the C-termini of Ras proteins function in plasma membrane binding (through both electrostatic binding of basic residues and hydrophobic binding of fatty acylated residues) [14], sequence variations in this region could affect the biological functions of the isoforms. Indeed, in transfected cells, human *Kras*-4A, but not *Kras*-4B, efficiently induced transformed foci and enabled anchorage-independent growth [15], [16]. Additional differences between the two isoforms regard their expression levels, which were found to be modulated during mouse embryogenesis and to vary in different tissues [17], [18]. Moreover, the ratio between *Kras*-4A and *Kras*-4B mRNA levels in lung tissue was higher in mouse strains susceptible to lung cancer than in resistant ones [17]. More recent evidence implicating the *Kras*-4A oncoprotein in lung tumorigenesis was provided by two studies in which mice expressing only the *Kras*-4B isoform had greater resistance to chemical carcinogenesis than did wild-type mice also expressing *Kras-4A* [19], [20].

Despite these observations, several aspects of the candidacy of *Kras* as the major effector of the *Pas1* locus in lung tumor susceptibility remain to be clarified. Wild-type alleles from susceptible and resistant mice code for the same protein; consequently, the mechanism by which *Kras* determines genetic susceptibility to lung tumorigenesis could, instead, depend on its overall expression level or on the ratio of its isoforms in normal lung tissue.

To address these points and to clarify the involvement of the two *Kras* isoforms, we studied *Kras* mRNA levels and germline variants using an established model of urethane-induced lung tumorigenesis in an advanced intercross population between A/J mice (tumor susceptible) and C57BL/6 mice (resistant). This model was previously instrumental in defining the *Pas1* locus based on the pattern of tumorigenesis in F2 intercross mice [1]. In the present study, we used F4 mice (of a new pedigree) in order to have a greater resolution power. After confirming *Pas1* as a quantitative trait locus (QTL) modulating lung tumor multiplicity in this pedigree, we performed a genome-wide association study to look for expression QTL modulating the levels of *Kras*-4A and *Kras*-4B transcripts in lung tumors and normal lung tissue. Our findings indicate that *Kras*-4A and, to some extent, also *Kras*-4B are modulated by *cis*-acting elements within the *Pas1* locus itself. These results underpin a possible causal relationship between germline variations and mRNA levels of the *Kras*-4A isoform, consequently influencing lung tumor susceptibility.

## Results

A/J and C57BL/6 mice were mated and bred to the fourth generation (ABF4) to establish a pedigree for the current study. Male ABF4 mice (n = 183) were treated with a single injection of urethane at 4 weeks of age to induce lung tumor formation, sacrificed 36 weeks later and assessed for lung tumor multiplicity (Nlung). Values of Nlung ranged from 0 (in 27 animals) to a maximum of 33.

**Figure 1:**
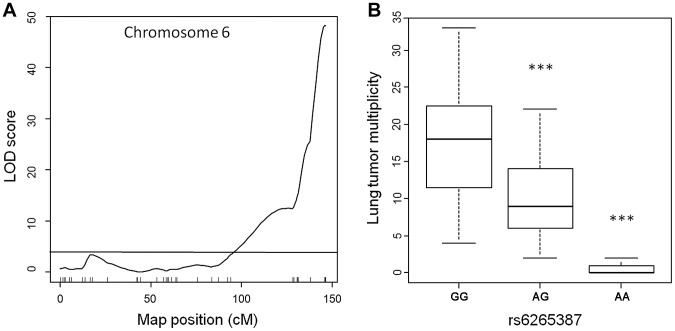
Lung tumor multiplicity in 183 urethane-treated male ABF4 mice is controlled by the *Pa*s*1* locus. (a) LOD score plot for chromosome 6 on which a quantitative trait locus (QTL) for lung tumor multiplicity (square root transformed values) mapped to the telomeric region. The QTL peak (LOD score = 48, phenotypic variance explained = 69%) overlapped with the *Pas1* locus. Tick marks show the position of 37 genotyped markers, including rs6265387 at the QTL peak. Horizontal line indicates the 95% LOD threshold. (b) Number of lung tumors per animal, grouped according to genotype at rs6265387. Mice homozygous for the A/J-derived allele (GG; n = 59) had more tumors than either heterozygous animals (n = 82) or mice homozygous for the C57BL/6-derived allele (AA; n = 42). ***P<1.0×10^−6^ versus the A/J-derived allele, ANOVA followed by Tukey's test for multiple comparisons. The line within each box represents the median; upper and lower edges of each box are 75^th^ and 25^th^ percentiles, respectively; upper and lower bars indicate the highest and lowest values less than one interquartile range from the extremes of the box.

To assess the genetic control of susceptibility to lung tumorigenesis in this pedigree, we performed genome-wide linkage analysis on the 183 urethane-treated ABF4 mice using Illumina SNP-arrays, which permit the genotyping of up to 1449 single nucleotide polymorphisms (SNPs). After quality control filtering, genotype data were obtained for 548 informative (polymorphic) non-redundant SNPs dispersed over the whole genome. Simple interval mapping was performed to detect QTLs associated with squared root-transformed values of Nlung. This analysis identified a single major locus in the telomeric region of chromosome 6 (**Figure 1A**). The QTL peak had a LOD score of 48.2 and was centered around marker rs6265387 (*Pthlh* gene region) near the *Pas1* locus; moreover, at the *Kras* 37-bp marker, located 2-Mb proximal to rs6265387 and distinguished from the SNP by 3 recombination events, the LOD score was almost identical (LOD = 48.0). The QTL accounted for 69% of the total phenotypic variance in the ABF4 population. When the mice were grouped according to genotype (**Figure 1B**), Nlung in the 59 animals homozygous for the A/J-derived allele (represented by a G at rs6265387) was 1.5-fold higher than that in the 82 heterozygous mice and ∼24-fold higher than that in the 42 mice homozygous for the C57BL/6-derived allele (represented by an A at rs6265387; *P*<1.0×10^−6^, ANOVA followed by Tukey's test for multiple comparisons).

These results, which confirm the key role of the *Pas1* locus in murine lung tumorigenesis, establish this new intercross population as suitable for studying *Pas1* function. In this cross, lung carcinogenesis can be considered a monogenic trait due to the overwhelming genetic effect of the *Pas1* locus. Therefore, we used this pedigree to test whether the *Pas1* locus exerts its effect on lung tumorigenesis through a modulation of *Kras* mRNA levels. To this aim, we performed linkage analyses to identify expression QTLs associating with the levels of *Kras*-4A and *Kras*-4B mRNA.

### *Pas1* is an expression QTL controlling *Kras*-4A transcript levels in lung tumors of urethane-treated mice

From 80 of the 183 ABF4 urethane-treated mice used in the genetic linkage analysis, we were able to resect a tumor specimen from lung tissue. This subgroup included 37 mice homozygous at rs6265387 for the A/J-derived allele (associated with tumor susceptibility), 34 heterozygous mice, and 9 mice homozygous for the C57BL/6 resistant allele that nevertheless had developed a lung tumor. Indeed, the intrinsic low susceptibility to lung tumorigenesis of the 42 animals homozygous for the C57BL/6-derived allele (only 9 of these mice developed a lung tumor) did not allow us to analyze a subgroup of the original population with a genotype ratio typical of such intercrosses (i.e., 1∶2∶1).

**Figure 2:**
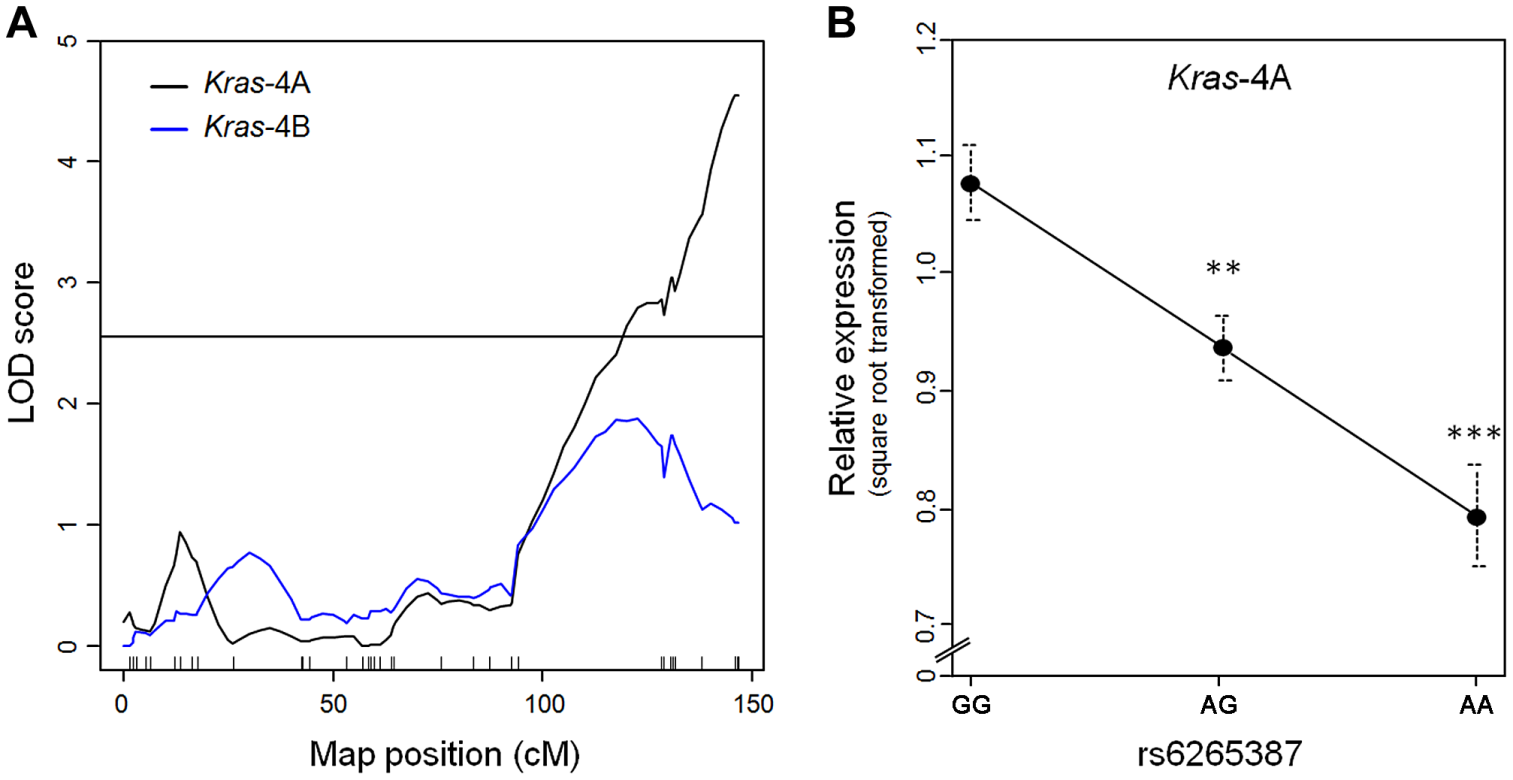
*Kras*-4A levels in lung tumors from 80 urethane-treated ABF4 mice are controlled by *Pas1* locus. (a) Expression QTL analysis of *Kras* transcripts showed that square-root-transformed levels of *Kras*-4A linked to the *Pas1* locus (LOD = 4.5). No significant linkage was observed for the *Kras*-4B mRNA isoform. Tick marks show the position of the genotyped markers in a recombinational map. Horizontal line at LOD = 2.55 marks the 95% threshold for significance. (b) Relative expression levels of the *Kras*-4A isoform according to rs6265387 genotype. Mice homozygous for the A/J-derived susceptible allele (GG, n = 37) had higher levels than either heterozygous animals (AG, n = 34) or mice homozygous for the C57BL/6 resistant allele (AA, n = 9). ***P<0.001, **P<0.01 vs. GG mice, ANOVA followed by Tukey's test for multiple comparisons. Values are means and SE.

RNA was extracted from each specimen and used in quantitative PCR to measure the levels of *Kras*-4A and -4B mRNA. Genome-wide linkage analysis for *Kras*-4A found a single expression QTL on chromosome 6 (peak LOD = 4.5), corresponding to the *Pas1* locus (**Figure 2A**). In contrast, no expression QTL was found for *Kras*-4B on any chromosome, including chromosome 6 where LOD scores remained below the threshold for significance. When the 80 mice were grouped according to the genotype at rs6265387, we found that *Kras*-4A mRNA was highest in mice homozygous for the A/J-derived susceptible allele (GG), intermediate in heterozygous (AG) animals, and lowest in mice homozygous for the C57BL/6 resistant allele (AA); these differences were significant (GG vs. AG, *P* = 3.2×10^−3^; GG vs. AA, *P* = 8.9×10^−5^; ANOVA followed by Tukey's test for multiple comparisons, **Figure 2B**). These results implicate *Kras*-4A in the mechanism of *Pas1*-dependent tumor formation.

### *Pas1*-dependent tumor susceptibility correlates with *Kras*-4A transcript levels in normal lung tissue

The genetic susceptibility to cancer is an intrinsic feature of normal tissue that, in urethane-treated mice, influences both the probability of tumor initiation and the number of tumors that develop. Consequently, it is likely that the fundamental elements underlying this phenomenon are present not only in tumors but also in normal tissue. To test this hypothesis, we attempted to validate in normal lung tissue the genetic linkage we observed in lung tumors. Therefore, we genotyped 111 untreated male ABF4 mice for nine markers on chromosome 6 spanning from 96.7 Mb to 148.3 Mb. These markers include 8 SNPs and one 37-bp sequence variation that in C57BL/6 mice occurs as a tandem repeat; this insertion mutation is located in the second intron of *Kras* [21]. Additionally, we assayed lung mRNA from these mice for the two *Kras* isoforms, and examined the association of mRNA levels with genotype.

**Figure 3:**
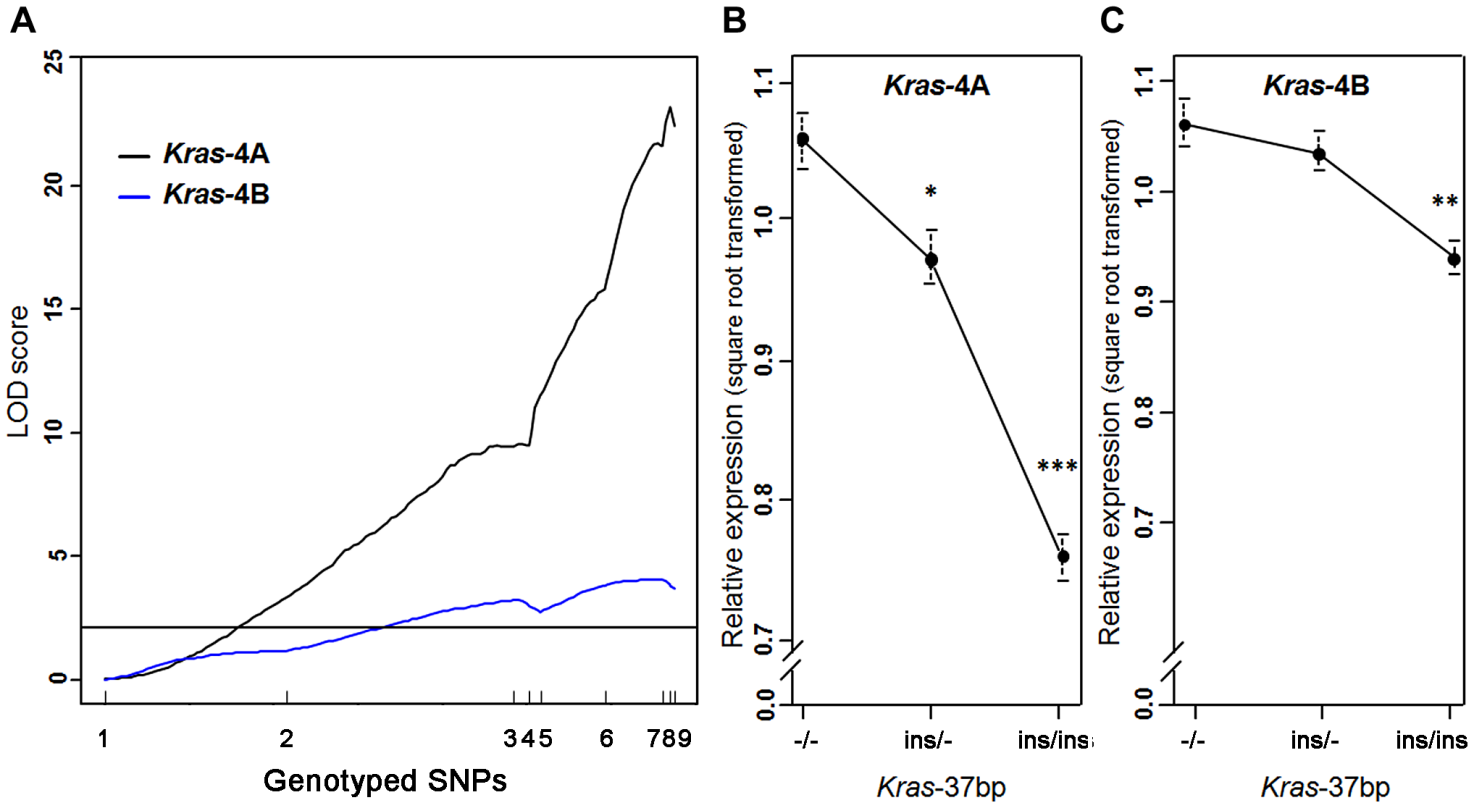
*Kras*-4A expression is highly controlled by *Pas1* locus in normal lung. (a) Genetic linkage analysis of expression levels (square root transformed) of two *Kras* transcripts in 111 untreated ABF4 mice showed that the *Kras*-4A isoform was strongly linked to the *Pas1* locus (LOD score = 23.2) whereas the *Kras*-4B isoform showed a weaker linkage (LOD score = 4.1). Horizontal line at LOD = 2.13 marks the threshold for significance. Tick marks show the position, in a recombinational map, of the nine genotyped markers spanning from chromosome 6 position 96.7 Mb to 148.3 Mb. (b, c) Expression levels of *Kras*-4A and -4B in normal lung tissue, by genotype for the 37-bp insertion mutation common to both isoforms. The A/J-derived susceptible allele is negative (-) for the insertion whereas the C57BL/6 allele is positive (ins); 30 mice were -/-, 52 ins/-, and 29 ins/ins. (b) For *Kras*-4A, *P* = 9.7×10^−14^, ANOVA. Tukey's test for multiple comparisons, *P = 0.01, ***P = 7.4×10^−14^ vs. -/-. (c) For *Kras*-4B, *P* = 9.7×10^−5^, ANOVA. Tukey's test for multiple comparisons, **P = 1.5×10^−4^ vs. -/-. Values are means and SE.

A strong linkage was found between the level of *Kras*-4A mRNA and genotyped markers in the *Pas1* locus, describing a LOD curve with a peak of 23.2 (**Figure 3A**); this expression QTL explained 62% of the phenotypic variance. A much weaker, yet statistically significant linkage for the *Kras*-4B transcript was observed (maximum LOD score = 4.1). These results confirm and strengthen the major role of *Kras*-4A isoform found in tumors (**Figure 2**). We then grouped the mice according to genotype at the *Kras* 37-bp insertion to examine the relationship between *Kras* mRNA levels and the number of inherited A/J-derived alleles of the *Pas1* locus. A strong association was found for *Kras*-4A levels (*P* = 9.7×10^−14^, ANOVA; square-root-transformed values are shown in **Figure 3B**): mice with two A/J-derived alleles (i.e. no insertion, -/-) had on average 1.9-times more transcript than did mice receiving two C57BL/6-derived alleles (ins/ins), and heterozygous mice had intermediate levels. In contrast, the linkage between *Pas1* genotype and *Kras*-4B transcript levels was weaker (*P* = 9.7×10^−5^, ANOVA, **Figure 3C**).

**Figure 4:**
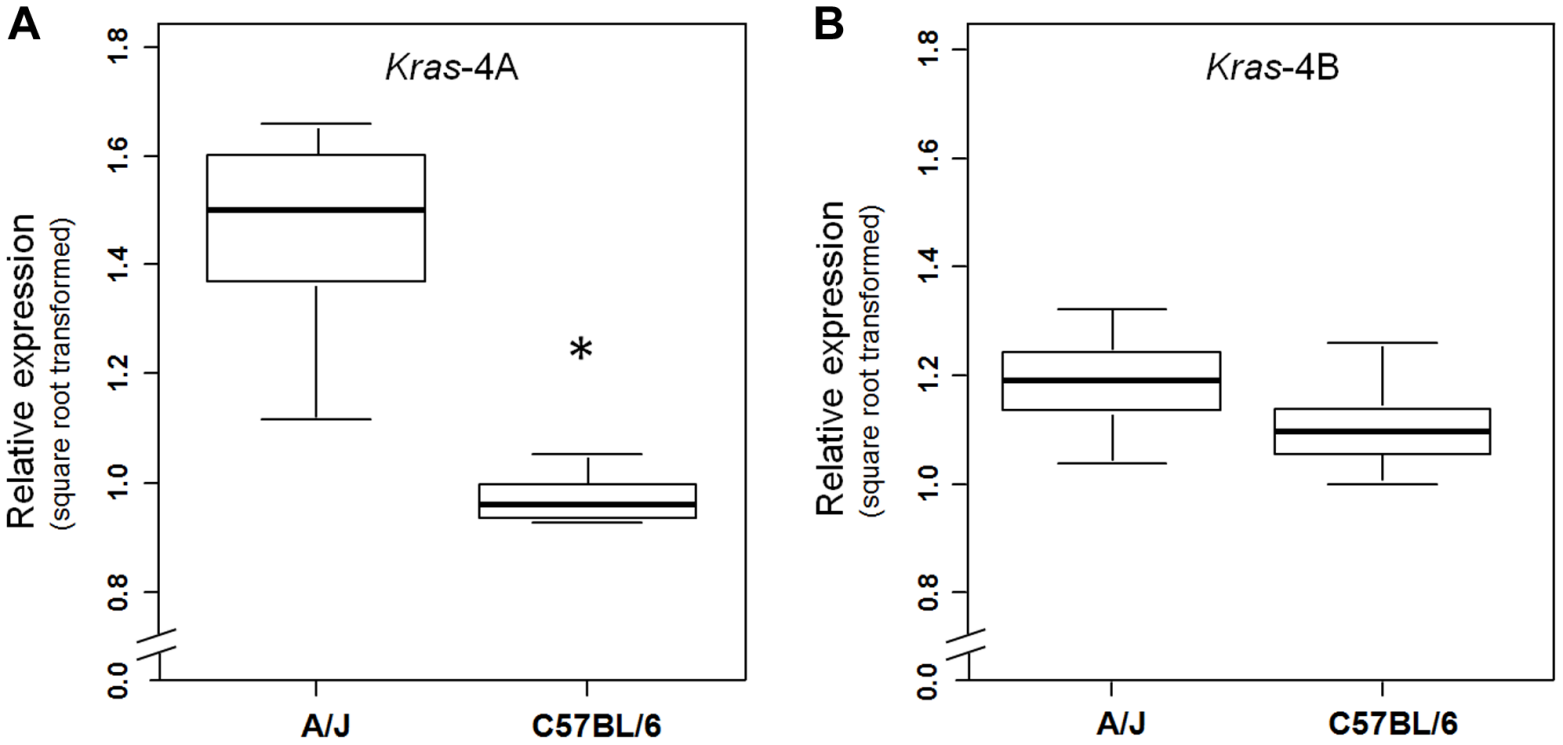
*Kras*-4A is expressed at higher levels in susceptible (A/J) than resistant (C57BL/6) strains. Expression levels of *Kras*-4A (a) and *Kras*-4B (b) isoforms were measured by qPCR in normal lung of 11 A/J and 11 C57BL/6 mice. * *P* = 7.1×10^−5^, ANOVA. The line within each box represents the median of square root transformed values; upper and lower edges of each box are 75^th^ and 25^th^ percentiles, respectively; upper and lower bars indicate the highest and lowest values less than one interquartile range from the extremes of the box.

We then examined whether this specific genetic control exerted by the *Pas1* locus was also present in normal lung of the parental inbred strains A/J and C57BL/6 (**Figure 4**). The median level of the *Kras*-4A transcript in A/J (tumor-susceptible) mice was 2.3-fold higher than that of C57BL/6 (resistant) mice (*P* = 6.0×10^−9^, ANOVA; square-root-transformed values are shown in **Figure 4A**). In contrast, the *Kras*-4B levels were not significantly different between mouse strains (**Figure 4B**).

Altogether, these results indicate that the A/J-derived allele of the *Pas1* locus, which confers susceptibility to lung tumorigenesis, is also associated with higher steady-state levels of the *Kras*-4A splice variant. These observations suggest that subtle modulation of *Kras*-4A mRNA production or stability may be the key effector of *Pas1*-controlled lung tumor susceptibility, possibly via a *cis*-acting element within *Pas1* itself.

### Differential allelic expression of *Kras*-4A is due to sequence variants in *cis*-regulatory elements

To test whether the allele-specific levels of *Kras* isoforms are attributable to variations in *cis*-regulatory elements also mapping in the *Pas1* locus, we examined the possibility of differential allelic expression. For this analysis, we considered two SNPs (rs29968550 and rs30022167, mapping in a region of *Kras* mRNA common to the two isoforms) whose genotypes permitted us to distinguish the A/J- from the C57BL/6J-derived allele. By pyrosequencing, we determined the frequencies of the two alleles in *Kras*-4A and -4B cDNA from normal lung tissue of 20 heterozygous untreated ABF4 mice and from lung tumor specimens of 15 heterozygous treated mice and in genomic DNA from the same animals, used as control for the amplification efficiency of the two alleles (**Figure S1**). Indeed, when allelic imbalance is tested, uneven amplification unrelated to differential transcription may occur in some of the assays. Therefore, the experimentally obtained allelic ratio for genomic DNA, which in principle is equal to 1 in heterozygotes, is used as the baseline to evaluate the ratios observed for cDNAs.

**Figure 5:**
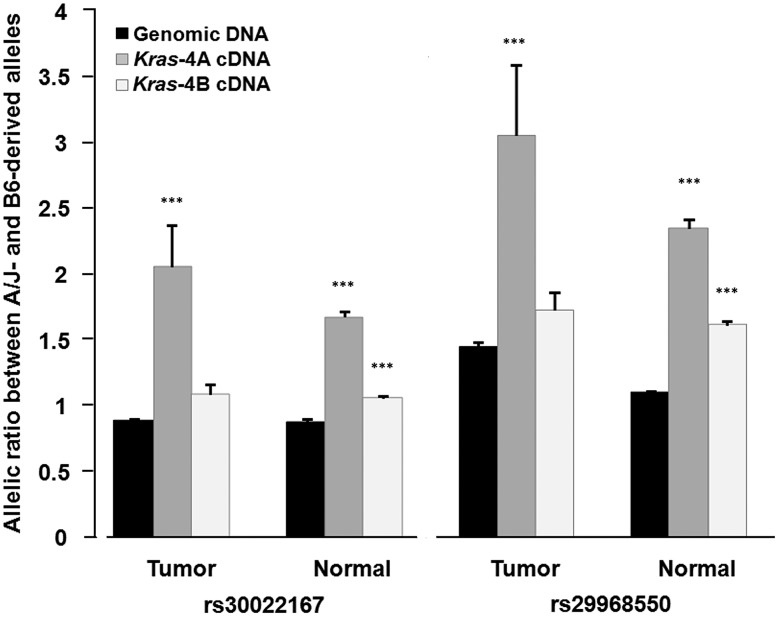
Differential allelic expression of *Kras*-4A isoform indicates the existence of functional polymorphisms in *Kras* gene. Allelic ratios were determined by pyrosequencing for rs30022167 and rs29968550, which map in a region of the *Kras* 3′-UTR common to both isoforms, on genomic DNA and cDNA from normal lung tissue (n = 20) and lung tumor specimens (n = 15) from ABF4 heterozygous mice. Values are mean and SE; *** P<0.001, two-sided Welch's t test (see **Table 1** for complete data).

Based on the results for both SNPs, comparing the mean allelic ratios for *Kras*-4A cDNA with those of the corresponding genomic DNA, we observed a ∼2-fold higher expression of the A/J-derived allele in *Kras*-4A cDNA in both tumor and normal tissue indicating a higher steady state of this isoform associated with the A/J-derived allele, possibly due to either a higher production rate or a lower degradation rate (**Figure 5**). For *Kras*-4B, the mean allelic ratios for cDNA from tumors were similar to those for genomic DNA, meaning equal production from both alleles; in normal tissue, however, a slightly higher ratio for *Kras*-4B cDNA relative to genomic DNA was observed (**Figure 5**).

In the statistical analysis, allelic ratios were log_10_-transformed to approximate a normal distribution before applying Welch's *t* test (**Table 1**). This analysis showed that the high allelic ratios for *Kras*-4A cDNA, observed at two SNPs and in both normal and tumoral lung tissue, are significantly different from those of genomic DNA (*P*<0.001, see **Table 1**). In the case of *Kras*-4B cDNA, the allelic ratios were significantly different from that of genomic DNA only for normal lung tissue (*P*<0.001). These results provide evidence for the differential allelic expression of *Kras* transcripts, with higher levels being produced from the A/J-derived allele in ABF4 intercross mice. These findings are compatible with the presence of allele-specific germline variations in *cis*-acting elements that mainly influence the selection or stability of the *Kras*-4A isoform.

**Table 1:** Differential allelic expression of *Kras* isoforms in lung tumor specimens and normal lung tissues from ABF4 intercross mice.

			Mean log_10_-transformed allelic ratio*			
SNP	Tissue	Isoform	cDNA	Genomic DNA	Difference (95% CI)	P value#
rs30022167	Tumor	Kras-4A	0.26	−0.05	0.31 (0.20 to 0.43)	3.7×10^−5^
		Kras-4B	0.01	−0.05	0.06 (−0.03 to 0.16)	0.17
	Normal	Kras-4A	0.22	−0.07	0.29 (0.26 to 0.32)	<2.2×10^−16^
		Kras-4B	0.02	−0.07	0.09 (0.06 to 0.12)	6.1×10^−8^
rs29968550	Tumor	Kras-4A	0.42	0.16	0.26 (0.13 to 0.39)	8.4×10^−4^
		Kras-4B	0.22	0.16	0.06 (−0.03 to 0.15)	0.19
	Normal	Kras-4A	0.37	0.04	0.33 (0.30 to 0.37)	<2.2×10^−16^
		Kras-4B	0.21	0.04	0.17 (0.15 to 0.20)	<2.2×10^−16^

* Mean of log_10_ transformed allelic ratios (A/J-derived allele/C57BL/6-derived allele) for 20 samples (normal tissue) or 15 samples (tumor tissue).

# Calculated on differences between cDNA and DNA allelic ratios using a two-tailed Welch's *t* test.

## Discussion

In this study, we verified the major role of the *Pas1* locus in modulating lung tumorigenesis in an ABF4 advanced intercross population, where the lung tumor multiplicity (Nlung) phenotype behaved as a monogenic trait under the control of the *Pas1* locus, explaining almost 70% of the phenotypic variance. In addition, we found that *Pas1* is an expression QTL because it controls the level of the *Kras*-4A isoform in urethane-induced lung tumors. This genetic control is an inherited trait, in that it was already present in normal lung tissue of untreated mice. Finally, we observed the preferential expression of *Kras*-4A from the A/J-derived allele in heterozygous mice, suggesting the existence of allele-specific germline variations in *cis*-acting elements that influence splicing bias, selection or stability of this isoform.

We carried out this genetic study in an ABF4 intercross since it was an effective way to accumulate, in a population of a relatively small size, 3-fold more recombination events than those possible in a conventional F2 intercross population of the same size [22]. Indeed, each ABF4 mouse underwent six informative meioses compared to the two informative meioses of an F2 mouse, thus providing improved resolution for mapping loci affecting strain-related phenotypes [22]. This methodological choice allowed us to obtain a sharp peak of linkage between *Pas1* and Nlung, with very high association (LOD score = 48 in 183 ABF4 mice). The high rate of recombination in this intercross was also useful for testing our hypothesis that *Kras* isoform expression is modulated by *Pas1*.

Animals homozygous for the C57BL/6-derived allele had low susceptibility to lung tumorigenesis and, therefore, rarely developed lung tumors after urethane treatment. Hence, the genetic class with the resistant phenotype was under-represented in the experiment designed to identify expression QTLs in tumors. Another difficulty that presented in our research was the known dysregulation of gene expression architecture in cancer, including mouse lung tumors [23], [24], which could have reduced the detectability of expression QTLs. Despite these limitations, we observed a significant linkage of the *Pas1* locus with mRNA levels of *Kras*-4A (LOD score = 4.5 in 80 mice) but not of *Kras*-4B.

In normal lung tissues from untreated mice, where all three genotypes were present at roughly the expected ratio and where no pathologic alterations could have played a confounding role, we observed that the *Kras*-4A isoform levels were again significantly linked with the *Pas1* locus (LOD score = 23.2 in 111 mice). In these untreated animals, the *Kras*-4B isoform also had significant linkage with *Pas1*, although the LOD score of 4.1 was only ∼17% that obtained for *Kras*-4A.

The differential allelic expression of *Kras*-4A and, to some extent, *Kras*-4B, in normal lung tissue and in lung tumor specimens, indicated the existence of regulatory polymorphisms located within the *Pas1* locus. These *cis*-acting elements have not yet been identified. However, since the *Kras* gene promoter has been shown to have similar activity in susceptible and resistant mice [25], it is conceivable that these *cis*-acting elements are located in other regions such as introns or the 3′-untranslated region of the *Kras* transcript. Regulatory elements in these regions can be expected to impact more on mRNA splicing and stability than on promoter activity. Several polymorphisms in non-coding regions of *Kras* have already been found in various mouse inbred strains and the functional roles of some of them have been investigated [21], [25], [26]; these studies, however, reached contrasting conclusions. Future investigations could focus on the targeted re-sequencing of the A/J-derived *Kras* gene and flanking regions, to search for not yet identified polymorphisms that affect splicing selection or mRNA stability of *Kras* isoforms. The candidate functional polymorphisms could then be tested by cloning into reporter vectors for transfection and assay of cell lines.

Overall, these results shed new light on the role of expression QTLs in mouse lung tumorigenesis, since they indicate that the genetic control exerted by *Pas1* mostly affects the expression of the *Kras*-4A transcript. These results point to the 4A isoform as the functional element of *Pas1*, the major locus modulating lung tumorigenesis in mice.

## Materials and Methods

### Ethics statement

All animals received humane care according to the criteria outlined in a protocol approved during the meeting board of December 21, 2006, by the institutional ethical committee for animal use (CESA) at the Fondazione IRCCS Istituto Nazionale dei Tumori.

### Mouse crosses, tissues, DNA and RNA

A pedigree of intercross mice was generated by mating lung tumor-susceptible A/J mice (A; 5 females) with lung tumor-resistant C57BL/6 mice (B; 5 males). From generation ABF2 to ABF4, male and female mice were individually labeled, randomly selected using a random number generator and bred, avoiding the pairing of siblings (20 female and 20 male ABF2 mice, and 55 female and 55 male ABF3 mice were mated). After weaning, ABF4 mice were sexed and ear-tagged, and a section of tail was collected for DNA extraction. Male ABF4 mice were then randomly assigned to two groups: untreated mice (n = 131) were raised under standard conditions, while 188 male mice were treated with a single intraperitoneal injection of urethane (1 g/kg body weight) at 4 weeks of age to induce the development of lung tumors [27].

To obtain normal lung tissue, 111 untreated AFB4 mice, 11 A/J and 11 C57BL/6 mice (all males) were anesthetized and killed at 16 weeks of age; lung lobes were isolated and frozen in liquid nitrogen. To obtain lung tumor tissue, the 183 urethane-treated mice were killed at 40 weeks of age; the chest was opened, the trachea was dissected to permit needle access, and the lungs were filled with 0.5 ml RNALater solution. Then, the lung lobes were removed, placed in RNALater, and kept overnight at 4°C degrees. The next day, lungs were examined for surface tumors. For each mouse, we recorded the total number of tumors (Nlung) and, whenever possible, resected one tumor of about 1–1.5 mm under a stereo-microscope. These tumor specimens were frozen and stored at −80°C degrees.

Genomic DNA was extracted from tail samples using the DNeasy Blood & Tissue Kit (Qiagen, Valencia, CA, USA) and quantified using Picogreen dsDNA Quantitation Kit (Invitrogen, Life Technologies, Paisley, UK). Total RNA was extracted from normal lung tissue and from lung tumor specimens using RNeasy Midi Kit (Qiagen), purified with RNeasy MinElute Cleanup (Qiagen), and quantified by spectrophotometry (ND-1000 spectrophotometer, NanoDrop, Wilmington, DE, USA). RNA integrity was verified using the RNA 6000 Nano Assay Kit (Agilent Technologies, Palo Alto, CA, USA); the mean RIN value was 8.9 (SD = 0.8; range, 6.7 to 9.9).

### Genome-wide SNP and *Pas1* locus genotyping

Genomic DNA from urethane-treated ABF4 mice was used for genome-wide SNP genotyping carried out with the GoldenGate Genotyping Assay according to the manufacturer's protocol (Illumina, San Diego, CA, USA), using the Mouse MD Linkage Panel representing 1449 mouse loci.

Additionally, the *Pas1* locus in untreated ABF4 mice was genotyped for nine genetic markers, including eight SNPs (rs6349084, rs33863668, rs31000839, rs31005929, rs13479063, rs33893742, rs13479082, rs3711088) and a 37-bp tandem repeat in *Kras* [21]. Briefly, a genomic fragment surrounding each marker was PCR-amplified in reactions containing 30 ng genomic DNA, 1 U AmpliTaq Gold (Applied Biosystems, Life Technologies), 1× AmpliTaq Gold buffer (Applied Biosystems, Life Technologies), 1.5 mM MgCl_2_, 200 µM dNTPs and 5 pmol of each of a pair of specific primers (**Table S1**) in a total volume of 25 µl. To genotype the eight SNPs, PCR products were pyrosequenced on a PyroMark Q96 ID system running PyroMark Q96 ID Software (Qiagen). To genotype the *Kras* 37-bp repeat, PCR amplicons were analyzed by 3% agarose gel electrophoresis for fragment size.

### Quantitative real-time PCR

RNA from normal lung (1 µg) or from lung tumor specimens (0.5 µg) was used to synthesize cDNA by reverse transcription using the Transcriptor First Strand cDNA Synthesis Kit (Roche, Basel, Switzerland). *Kras-4A* and -*4B* transcript levels in the cDNA were measured in quantitative PCR (qPCR) assays using Fast SYBR Green PCR Master Mix (Applied Biosystems) and 300 nM intron-spanning primers (**Table S1**). Hypoxanthine phosphoribosyltransferase 1 (*Hprt1*, **Table S1**) was used as reference gene. Relative expression levels were calculated using the comparative Ct method using one of the synthesized cDNA samples as calibrator.

### Detection of differential allelic expression of *Kras*-4A and -4B transcripts

The allelic expression of *Kras*-4A and *Kras*-4B isoforms was analyzed taking into consideration two SNPs in the 3′-UTR region of the *Kras* gene (**Figure S1**): rs30022167 (A or C, for the A/J- or C57BL/6-derived allele, respectively) and rs29968550 (A or G, for the A/J- or C57BL/6-derived allele, respectively). Among the animals heterozygous for markers at the *Pas1* locus, we selected 20 mice from the untreated group and 15 from the urethane-treated group, from which we already had genomic DNA. In addition, from these animals, we also had cDNA from normal lung (n = 20) or from tumor specimens (n = 15), having been reverse-transcribed for the qPCR experiments. For each *Kras* isoform, we carried out a first amplification step on the cDNA samples using a forward primer located in exon 4A (*Kras*-4A) or in the junction between exon 3 and exon 4B (*Kras*-4B) and a common reverse primer located in the 3′-UTR region of *Kras* gene downstream of rs30022167 and rs29968550 (**Table S1** and **Figure S1**). The resulting PCR product as well as a sample of genomic DNA from the same animal were PCR-amplified using SNP-specific primers. These PCR products were pyrosequenced on a PyroMark Q96 ID system running PyroMark Q96 ID Software (Qiagen). For each SNP, the proportions of the two alleles present in each sample (genomic DNA, *Kras*-4A cDNA and *Kras*-4B cDNA from untreated and tumor-bearing mice) were determined from pyrogram peak heights, and allelic ratios (A/J-derived allele/C57BL/6-derived allele) were calculated.

### Statistical analyses

Nlung values and mRNA levels were square-root transformed to improve the normality of distribution. The transformed values and genotype data were analyzed by simple interval mapping using R/qtl [28] to identify QTLs. LOD scores were considered significant if greater than the 95% LOD threshold (α = 0.05), calculated by 10,000 permutations. The percent phenotypic variance explained by a given QTL was calculated from the LOD score by the following formula: R^2^ = [1−10^(−2LOD/n)^], where n is the sample size [28].

Differences between genotype groups were analyzed by one-way ANOVA followed by Tukey's test for multiple comparisons. Log_10_-transformed allelic ratios were compared between cDNA and genomic DNA using a two-tailed Welch's *t* test. Data were considered significant when *P*<0.01.

## Supporting Information

Figure S1 Experimental design for measuring the allelic expression of *Kras* isoforms. (a) First amplification step on cDNA was carried out using a forward primer located in exon 4A (green arrow) for the amplification of *Kras*-4A, or located in the junction between exon 3 and exon 4B (violet arrow) for *Kras*-4B, and a common reverse primer (red arrow) located in the 3′-UTR region of the *Kras* gene downstream of rs29968550 (blue triangle) and rs30022167 (orange triangle). (b) For pyrosequencing, SNP-containing fragments were amplified from the PCR amplicons obtained in the previous step and from genomic DNA of the same animals, using SNP-specific forward and reverse primers (blue arrows for rs29968550 and orange arrows for rs30022167) and a sequencing primer (dashed blue arrow for rs29968550 and dashed orange arrow for rs30022167).
(TIF)

Table S1 Primers used for PCR, genotyping and detection of differential allele expression (DAE).
(DOC)

## References

[1] Gariboldi M, Manenti G, Canzian F, Falvella FS, Radice MT, et al (1993) A major susceptibility locus to murine lung carcinogenesis maps on chromosome 6. Nature Genet 3: 132–136. 8499946 10.1038/ng0293-132

[2] Manenti G, Dragani TA (2005) Pas1 haplotype-dependent genetic predisposition to lung tumorigenesis in rodents: A meta-analysis. Carcinogenesis 26: 875–882. 15471897 10.1093/carcin/bgh299

[3] Manenti G, Galbiati F, Giannì Barrera R, Pettinicchio A, Acevedo A, et al (2004) Haplotype sharing suggests that a genomic segment containing six genes accounts for the pulmonary adenoma susceptibility 1 (Pas1) locus activity in mice. Oncogene 23: 4495–4504. 15064703 10.1038/sj.onc.1207584

[4] Wang M, Lemon WJ, Liu G, Wang Y, Iraqi FA, et al (2003) Fine mapping and identification of candidate pulmonary adenoma susceptibility 1 genes using advanced intercross lines. Cancer Res 63: 3317–3324. 12810665

[5] Wang M, Futamura M, Wang Y, You M (2005) Pas1c1 is a candidate for the mouse pulmonary adenoma susceptibility 1 locus. Oncogene 24: 1958–1963. 15688036 10.1038/sj.onc.1208295

[6] Liu P, Wang Y, Vikis H, Maciag A, Wang D, et al (2006) Candidate lung tumor susceptibility genes identified through whole-genome association analyses in inbred mice. Nat Genet 38: 888–895. 16862160 10.1038/ng1849

[7] King PD, Lubeck BA, Lapinski PE (2013) Nonredundant functions for ras GTPase-activating proteins in tissue homeostasis. Sci Signal 6: re1. 23443682 10.1126/scisignal.2003669 PMC5483993

[8] You M, Candrian U, Maronpot RR, Stoner GD, Anderson MW (1989) Activation of the ki-*ras* protooncogene in spontaneously occurring and chemically induced lung tumors of the strain A mouse. Proc Natl Acad Sci USA 86: 3070–3074. 2654935 10.1073/pnas.86.9.3070 PMC287066

[9] Re FC, Manenti G, Borrello MG, Colombo MP, Fisher JH, et al (1992) Multiple molecular alterations in mouse lung tumors. Mol Carcinog 5: 155–160. 1554414 10.1002/mc.2940050211

[10] Johnson L, Mercer K, Greenbaum D, Bronson RT, Crowley D, et al (2001) Somatic activation of the K-ras oncogene causes early onset lung cancer in mice. Nature 410: 1111–1116. 11323676 10.1038/35074129

[11] Meuwissen R, Linn SC, van d V, Mooi WJ, Berns A (2001) Mouse model for lung tumorigenesis through cre/lox controlled sporadic activation of the K-ras oncogene. Oncogene 20: 6551–6558. 11641780 10.1038/sj.onc.1204837

[12] Zhang Z, Wang Y, Vikis HG, Johnson L, Liu G, et al (2001) Wildtype Kras2 can inhibit lung carcinogenesis in mice. Nat Genet 29: 25–33. 11528387 10.1038/ng721

[13] To MD, Perez-Losada J, Mao JH, Hsu J, Jacks T, et al (2006) A functional switch from lung cancer resistance to susceptibility at the Pas1 locus in Kras2LA2 mice. Nat Genet 38: 926–930. 16823377 10.1038/ng1836 PMC4461000

[14] Prior IA, Hancock JF (2012) Ras trafficking, localization and compartmentalized signalling. Semin Cell Dev Biol 23: 145–153. 21924373 10.1016/j.semcdb.2011.09.002 PMC3378476

[15] Voice JK, Klemke RL, Le A, Jackson JH (1999) Four human ras homologs differ in their abilities to activate raf-1, induce transformation, and stimulate cell motility. J Biol Chem 274: 17164–17170. 10358073 10.1074/jbc.274.24.17164

[16] Plowman SJ, Arends MJ, Brownstein DG, Luo F, Devenney PS, et al (2006) The K-ras 4A isoform promotes apoptosis but does not affect either lifespan or spontaneous tumor incidence in aging mice. Exp Cell Res 312: 16–26. 16271715 10.1016/j.yexcr.2005.10.004

[17] Wang Y, You M, Wang Y (2001) Alternative splicing of the K-ras gene in mouse tissues and cell lines. Exp Lung Res 27: 255–267. 11293328 10.1080/019021401300054028

[18] Pells S, Divjak M, Romanowski P, Impey H, Hawkins NJ, et al (1997) Developmentally-regulated expression of murine K-ras isoforms. Oncogene 15: 1781–1786. 9362444 10.1038/sj.onc.1201354

[19] To MD, Wong CE, Karnezis AN, Del Rosario R, Di Lauro R, et al (2008) Kras regulatory elements and exon 4A determine mutation specificity in lung cancer. Nat Genet 40: 1240–1244. 18758463 10.1038/ng.211 PMC2654781

[20] Patek CE, Arends MJ, Wallace WA, Luo F, Hagan S, et al (2008) Mutationally activated K-ras 4A and 4B both mediate lung carcinogenesis. Exp Cell Res 314: 1105–1114. 18062963 10.1016/j.yexcr.2007.11.004

[21] Chen B, Johanson L, Wiest JS, Anderson MW, You M (1994) The second intron of the K-*ras* gene contains regulatory elements associated with mouse lung tumor susceptibility. Proc Natl Acad Sci USA 91: 1589–1593. 8108449 10.1073/pnas.91.4.1589 PMC43205

[22] Darvasi A, Soller M (1995) Advanced intercross lines, an experimental population for fine genetic mapping. Genetics 141: 1199–1207. 8582624 10.1093/genetics/141.3.1199 PMC1206841

[23] Bonner AE, Lemon WJ, Devereux TR, Lubet RA, You M (2004) Molecular profiling of mouse lung tumors: Association with tumor progression, lung development, and human lung adenocarcinomas. Oncogene 23: 1166–1176. 14647414 10.1038/sj.onc.1207234

[24] Melkamu T, Zhang X, Tan J, Zeng Y, Kassie F (2010) Alteration of microRNA expression in vinyl carbamate-induced mouse lung tumors and modulation by the chemopreventive agent indole-3-carbinol. Carcinogenesis 31: 252–258. 19748927 10.1093/carcin/bgp208

[25] Jones-Bolin SE, Johansson E, Palmisano WA, Anderson MW, Wiest JS, et al (1998) Effect of promoter and intron 2 polymorphisms on murine lung K-ras gene expression. Carcinogenesis 19: 1503–1508. 9744549 10.1093/carcin/19.8.1503

[26] Timofeeva OA, Gorshkova EV, Levashova ZB, Kobzev VF, Filipenko ML, et al (2002) Pulmonary carcinogenesis susceptibility-associated single-nucleotide polymorphisms in K-ras intron 2 affect the binding of factor gata-6 but not gene expression. Mol Biol (Mosk) 36: 817–824. 12391845

[27] Shimkin MB, Stoner GD (1975) Lung tumors in mice: Application to carcinogenesis bioassay. Adv Cancer Res 21: 1–58. 1108612 10.1016/s0065-230x(08)60970-7

[28] Broman KW, Wu H, Sen S, Churchill GA (2003) R/qtl: QTL mapping in experimental crosses. Bioinformatics 19: 889–890. 12724300 10.1093/bioinformatics/btg112

