# Supplementary figures and images for: Mouse Pulmonary Adenoma Susceptibility 1 Locus Is an Expression QTL Modulating Kras-4A
Source: PLoS Genet. 2014 Apr 17;10(4):e1004307. doi: 10.1371/journal.pgen.1004307 (PMC3990522; doi:10.1371/journal.pgen.1004307)

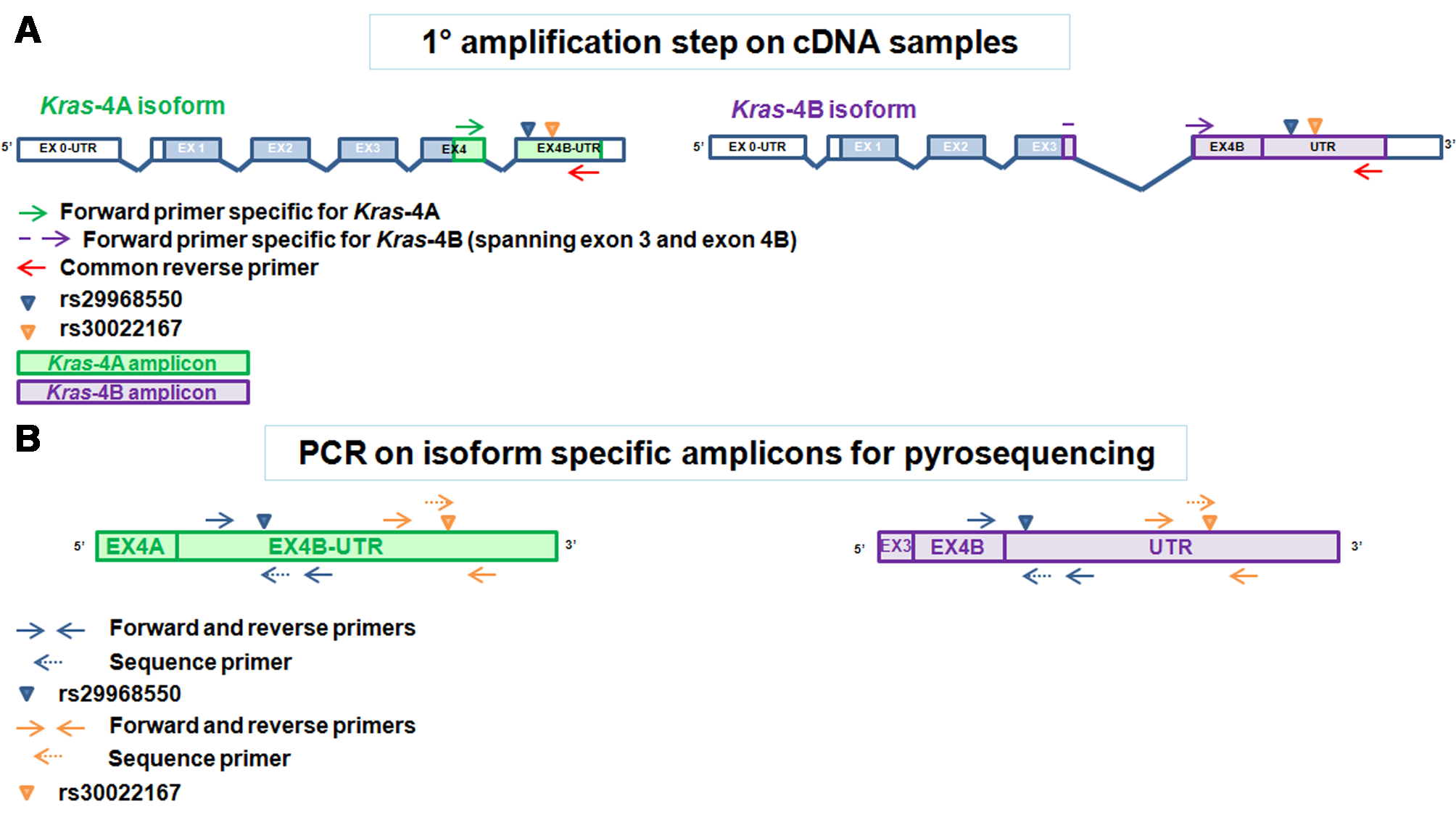

Supplement: Figure S1 — Experimental design for measuring the allelic expression of Kras isoforms. (a) First amplification step on cDNA was carried out using a forward primer located in exon 4A (green arrow) for the amplification of Kras-4A, or located in the junction between exon 3 and exon 4B (violet arrow) for Kras-4B, and a common reverse primer (red arrow) located in the 3′-UTR region of the Kras gene downstream of rs29968550 (blue triangle) and rs30022167 (orange triangle). (b) For pyrosequencing, SNP-containing fragments were amplified from the PCR amplicons obtained in the previous step and from genomic DNA of the same animals, using SNP-specific forward and reverse primers (blue arrows for rs29968550 and orange arrows for rs30022167) and a sequencing primer (dashed blue arrow for rs29968550 and dashed orange arrow for rs30022167). (TIF) [file pgen.1004307.s001.tif]
