# Supplementary material for: Mouse Pulmonary Adenoma Susceptibility 1 Locus Is an Expression QTL Modulating Kras-4A
Source: PLoS Genet. 2014 Apr 17;10(4):e1004307. doi: 10.1371/journal.pgen.1004307 (PMC3990522; doi:10.1371/journal.pgen.1004307)
Supplement: Table S1 — Primers used for PCR, genotyping and detection of differential allele expression (DAE). (DOC) [file pgen.1004307.s002.doc]

| **Table S1. Primers used for PCR, genotyping and detection of differential allele expression (DAE).** | | | | | |
| --- | --- | --- | --- | --- | --- |
| SNP ID or gene symbol | Chromosome 6 position (Mb) ^§^ | Forward primer ^#^ (5' -> 3') | Reverse primer ^#^ (5' -> 3') | Sequence primer (5' -> 3') | Use |
| rs6349084 * | 96747604 | cccttccttgctttgagaaaaac | Bio-cccaagagagattcctgaaaag | tgagactgagtccacca | PCR and pyrosequencing |
| rs33863668 * | 121669828 | Bio-ttggcttatccattatgagatctg | ttctgctgtgttgtggaacttc | catttctaaatatccactcc | PCR and pyrosequencing |
| rs31000839 * | 126927827 | catgctgggacagagtccattagt | Bio-caggaacatctgggaaattctttg | ggaacagactctggttttaa | PCR and pyrosequencing |
| rs31005929 * | 130368419 | Bio-ggttcacgctgctaactgg | tgaggtttgactaagggagttcag | gtacaaaagcttgcacat | PCR and pyrosequencing |
| rs13479063 * | 136390263 | aataagccaccactgacaagc | Bio-ttcctcatggcttaaggtcac | cttgcacactgctcc | PCR and pyrosequencing |
| rs33893742 * | 140007052 | Bio-gattcattccaccccattcag | ctcattgtagcctgggccact | tgtgtgctttggaaac | PCR and pyrosequencing |
| rs13479082 * | 144036772 | tccgtgtgcaaacatgtcc | Bio-ttcactttgggccctgtagttttt | tcacggtgacgcttc | PCR and pyrosequencing |
| *Kras* 37bp repeat * | 145216699 | gatggcatcttggaccttactc | agtctgcgtgcgcttgtaa |  | PCR |
| rs3711088 * | 148311947 | aggctgtggcttttgacact | Bio-ggatggcatgtgtgtaccact | tgacacttctctgtcaat | PCR and pyrosequencing |
| *Hprt* | NA | cgtcgtgattagcgatgatga | ccaaatcctcggcataatgatt |  | Quantitative PCR |
| *Kras*-4A | NA | cagcaaagacaagacagagagtgg | cttctttgctgatttttttcaatctgtac |  | Quantitative PCR |
| *Kras*-4B | NA | acaggctcaggagttagcaagga | aaggcatcgtcaacaccctgtc |  | Quantitative PCR |
| *Kras*-4A | NA | ttggtgagagagatccgaca | tgcctacgcctctgaaagat |  | cDNA PCR before DAE experiment |
| *Kras*-4B | NA | agcaaagacaagacagggtgt | tgcctacgcctctgaaagat |  | cDNA PCR before DAE experiment |
| rs29968550 | 145220160 | Bio-catccctgctctgtgtccatctac | tgcctacgcctctgaaagatgat | aggacacgctggacg | Genotyping, DAE |
| rs30022167 | 145220208 | cctgctctgtgtccatctactcat | Bio-acccctcagtgtccagtgaag | cattagcagtcaatcgc | Genotyping, DAE |
| ^§^ GRCm38 Assembly, Ensembl. ^#^ Bio, biotin. * Genetic markers represented in Figure 3 with number 1 to 9. NA, not applicable | | | | | |
